# Qualitative evaluation of factors influencing adherence to virtual exercise programs for people with physical disabilities

**DOI:** 10.3389/fresc.2024.1470630

**Published:** 2024-10-11

**Authors:** Madison Mintz, Christine Ferguson, Leigh Anne Bray Dayton, Jereme Wilroy, James H. Rimmer

**Affiliations:** ^1^ Department of Physical Therapy, School of Health Professions, University of Alabama at Birmingham, Birmingham, AL, United States; ^2^ Department of Nutrition Sciences, School of Health Professions, University of Alabama at Birmingham, Birmingham, AL, United States; ^3^ Capstone College of Nursing, University of Alabama, Tuscaloosa, AL, United States; ^4^ Department of Physical Medicine and Rehabilitation, Heersink School of Medicine, University of Alabama at Birmingham, Birmingham, AL, United States; ^5^ School of Health Professions, University of Alabama at Birmingham, Birmingham, AL, United States

**Keywords:** qualitative evaluation, people with physical disabilities, community programs, virtual exercise, program adherence, social cognitive theory (SCT)

## Abstract

Virtual community-based programming for people with disabilities has become a popular method for advocating for health promotion, specifically exercise, for people with disabilities (PWD). Using theoretical frameworks to better understand the perspective of PWD who participate in virtual exercise programs allows strategies of implementation following completion of virtual exercise programs. The objective of this study was to examine the effect adherence had on perceptions, experiences, and post-program exercise maintenance in participants with disabilities. Eight qualitative interviews were conducted in highly adherent participants using the Social Cognitive Theory (SCT). Interviews were recorded on Zoom, transcribed using Microsoft 365, and analyzed using NVivo software. Data were analyzed by the primary author and an independent coder to increase rigor and reduce bias. Thirty-five unique codes were generated from transcribed interviews. Member-checking was employed to increase internal validity; 100% of participants agreed with the findings. Results demonstrate an overall positive experience in the virtual exercise program, noting specific facilitators (i.e., knowledgeable instructor, program provided equipment, etc.) and barriers (i.e., limited physical space at home to exercise, other participant's attitudes, etc.) of participating. Impressionably, 100% of participants maintained exercise following their time within the virtual exercise program.

## Introduction

There are approximately one in eight individuals living with a physical or mobility disability in the United States (U.S.) as reported by the Centers for Disease Control and Prevention (CDC) (1, 2). Of this approximately 12% of the population, data suggest that unhealthy lifestyle concerns, such as obesity (41.6%), smoking (21.9%), heart diseases (9.6%), and diabetes (15.9%) are prominent and likely to occur up to 55% more within the disability demographic (1–3). Within the last decade, a significant shift has been made to prioritize health promotion and maintenance strategies specifically to and for people with disabilities (PWD) (3, 4). However, little to no data exists that illustrates the number of PWD who are recruited or informed about such programs or how many health promotion programs exist for PWD, although such programs are advertised through health agencies such as the CDC (4, 5). Several reports clearly outline health disparities and barriers to health enhancing behaviors encountered by PWD, such as lack of transportation, inaccessible facilities, and negative perceptions of healthcare providers (5–7). According to a recent retrospective surveillance survey that assessed behavioral risk factors among adults with mobility disabilities, approximately 40% of adults with mobility disabilities met one or both weekly physical activity guidelines of aerobic and muscle-strengthening exercises (7). Additionally, according to a recent global perspective analysis, PWD are 16%–62% less likely to meet physical activity guidelines and pose a higher risk of inactivity-related health problems when compared to people without disabilities (3, 8).

Nevertheless, with advocacy at the helm of health equity, diversity, and inclusion for PWD, recent literature has suggested an increase in accessible and inclusive opportunities for PWD (9). For example, community programs are peak areas of interest among participant involvement and research reporting. Community programs such as holistic health and wellness programs, support groups, and sports teams are becoming more popular and have several benefits (10–13). First, community programs offer inclusivity for a myriad of people, health conditions, geographic locations, demographics, and many more variables. Second, as a result of the Covid-19 pandemic, community programs for PWD and older adults are being offered through virtual platforms, such as Zoom (14–16). Finally, virtual participation has been found to alleviate barriers of participation such as travel and time constraints, cost to participate, environmental inaccessibility, and access to knowledgeable personnel (17).

Community programs are, however, benchmarked by success of the program itself, which is why adherence of participants is imperative (15, 16). For community programs delivered virtually to PWD, adherence and retention within the programs lead to successful participation, which leads to lifestyle implementation, leading ultimately to healthy decision-making behaviors (17, 18). One particular area of focus among virtually delivered community programs for PWD is exercise. Exercise is an important activity especially for people living with physical disabilities or mobility impairments, or for people with secondary or chronic health conditions (8, 14, 19–24). The literature consistently reports that exercise can serve as both preventative and restorative including alleviation of symptoms associated with inflammation, fatigue, poor nutrition, loneliness, depression, and sleep disorders (15, 16, 20, 25). Exercise may also produce mental and emotional benefits such as healthy decision-making behaviors that promote a generally healthier lifestyle (20–23). Therefore, virtual exercise programs overcome community barriers to participation and make it easier to participate in these online programs.

Several virtual exercise analyses for PWD confirm the benefits of performing physical activity and exercise within a comfortable setting, such as the participant's home. A recent study analyzed individuals with traumatic brain injury (TBI) in a 3-month tailored exercise program comparing two groups of participants’ adherence and compliance with an asynchronous (without trainer or exercise personnel) prescription program. One group (*n* = 10) performed aerobic exercises and the other group (*n* = 10) performed stretching and toning exercises. All participants completed the program, and the group that performed aerobic exercise maintained cardiorespiratory endurance compared to the group that performed stretching exercises (26). Another virtual exercise program evaluated people with Parkinson Disease (PD) who performed virtual physical and cognitive exercises with a trainer on Zoom twice each week for 16 weeks. Participants attended 81% of sessions and expressed their satisfaction and perceived benefits such as the program being useful for their current health management strategy (27). A third study examined the feasibility among individuals with spinal cord injury (SCI) during a home exercise program with a virtual trainer and an upper-body ergometer, where participants performed 30–45 min of exercise, three days/week for eight weeks. There was 100% adherence, increased aerobic capacity, higher volume of physical activity, and improved satisfaction with life. Participants also expressed the at-home intervention as advantageous for overcoming barriers to exercise typically experienced at a fitness facility (28). These studies provide evidence that virtual exercise programs are beneficial for health outcomes in PWD.

To further explore virtual interventions targeting health behavior among PWD, we conducted a qualitative study using Social Cognitive Theory (SCT) as our framework to describe perceptions and experiences of participants who joined a virtual holistic wellness program that included exercise for PWD. To explore relationships between exercise adherence and health behavior, we identified high-adherent participants from the exercise portion of the wellness program to interview regarding their perceptions and experiences of the program. This qualitative study aimed to answer the following question: *Does high-adherence within a virtually delivered program impact participants’ desire to implement learned physical activity strategies into their daily lives once the program is over?* To address this question, we examined the effect adherence had on perceptions, experiences, and post-program exercise maintenance in participants with disabilities through qualitative evaluation.

## Methods

### MENTOR program

MENTOR (Mindfulness, Exercise, Nutrition to Optimize Resilience) is a virtual community-based program offered to people with a range of disabilities, ages, ethnicities, and geographic locations. The MENTOR program is a free, completely online-delivered program for participants across the United States who have an existing disability or a recent diagnosis. MENTOR does not require a pre-requisite of engaging in mindfulness, exercise, or nutrition, rather the program focuses on teaching participants these strategies using a “from-the-ground-up” approach. To jumpstart engagement, participants receive a wellness box mailed to their physical address with all equipment needed to fully participate (i.e., wrist weights, resistance bands, notebook, yoga mat, etc.) The program is comprised of 3 core wellness domains, mindfulness, exercise, and nutrition, which teach self-care strategies. Each week, participants meet online via Zoom in a group class format to interact with a trained instructor to discuss elements of health and wellness. Class instructors include mindfulness, exercise, and nutrition coaches who have all been trained and certified in disability-specific content and motivational interviewing techniques to foster a inclusive and accessible online environment.

Mindfulness and nutrition have one 60-min class per week each across eight weeks, while exercise has two 60-min classes per week across eight weeks. The primary goal of mindfulness classes are to educate participants on how to focus on living in the present moment while learning coping strategies such as breathwork and learning how to manage stress, anxiety, nerves, etc. among others. Nutrition classes, taught by registered dieticians, focus on educating participants about proper nutrition and creating a healthy foundation for mindful eating. Exercise classes have live-instruction where the instructor exercises alongside participants, adapts movements in real-time, and educates about how movement positively impacts both the body and the mind. Participants create mindfulness, nutrition, and exercise goals and with direct access from their participant portal can set up one-on-one appointments with instructors, re-watch recorded sessions, and document their personal goals, milestones achieved, and experiences about their time within MENTOR.

In this program, participants are tasked with attending as many classes as they are able within their timeframe of participation and engaging with one another and their coaches. By the end of the eight-week program, participants are provided a plethora of information about how to practice better self-care within the areas of mindfulness, exercise, and nutrition. More information about the MENTOR program may be found online (https://www.nchpadconnect.org/mentor/) and elsewhere in literature (29–32).

### Sampling procedures and participants

The purpose of this study was to understand the factors that influence adherence to a virtual exercise program, MENTOR, for people with disabilities. Therefore, only the exercise class's adherence was accounted for when sampling for participants. It is important to note that participants who completed exercise could have also completed mindfulness and nutrition classes; however, only their completion and adherence of exercise classes were considered to remain within the scope of the purpose of the study. The primary researcher identified 419 participants who completed the virtual exercise classes between February 2022 through December 2023. Adherence was measured by calculating the number of classes each participant attended and dividing by sixteen, the total number of classes offered each eight-week wave of the program. Non-adherence was defined as attending zero virtual exercise classes, low adherence was defined as attending between 1 and 6 virtual exercise classes, moderate adherence was defined as attending between 7 and 11 virtual exercise classes, and high adherence was defined as attending 12 or more virtual exercise classes. Of 419 participants, 20% were non-adherers, 29% were low adherers, 18% were moderate adherers, and 33% were high adherers.

Purposive sampling was used to recruit high adherers. A complete list of high adherers was organized and randomized for interview selection. Participants were called to be informed about the purpose of the study and to schedule an interview upon interest. Due to the large time frame of program delivery (February 2022 to December 2023), participants were between seven and twenty-two months of having completed MENTOR.

### Conceptual framework

A behavioral explorative strategy which has been widely used in exercise programs targeting individuals with disabilities is the Social Cognitive Theory (SCT) (20–23). SCT is the interplay between behavioral decisions among PWD and strategies for lifestyle implementation. Using SCT allows us to better comprehend participant behaviors, health-related decisions, and programmatic strategies that need improving. SCT uses determinants of personal cognitive factors, socioenvironmental factors, and behavioral factors to determine outcome expectations and behavioral components such as self-efficacy and self-regulatory strategies. Both are major predictors of physical activity and strategies of change, as determined by Dr. Albert Bandura, the creator of SCT (20–23).

**Figure 1:**
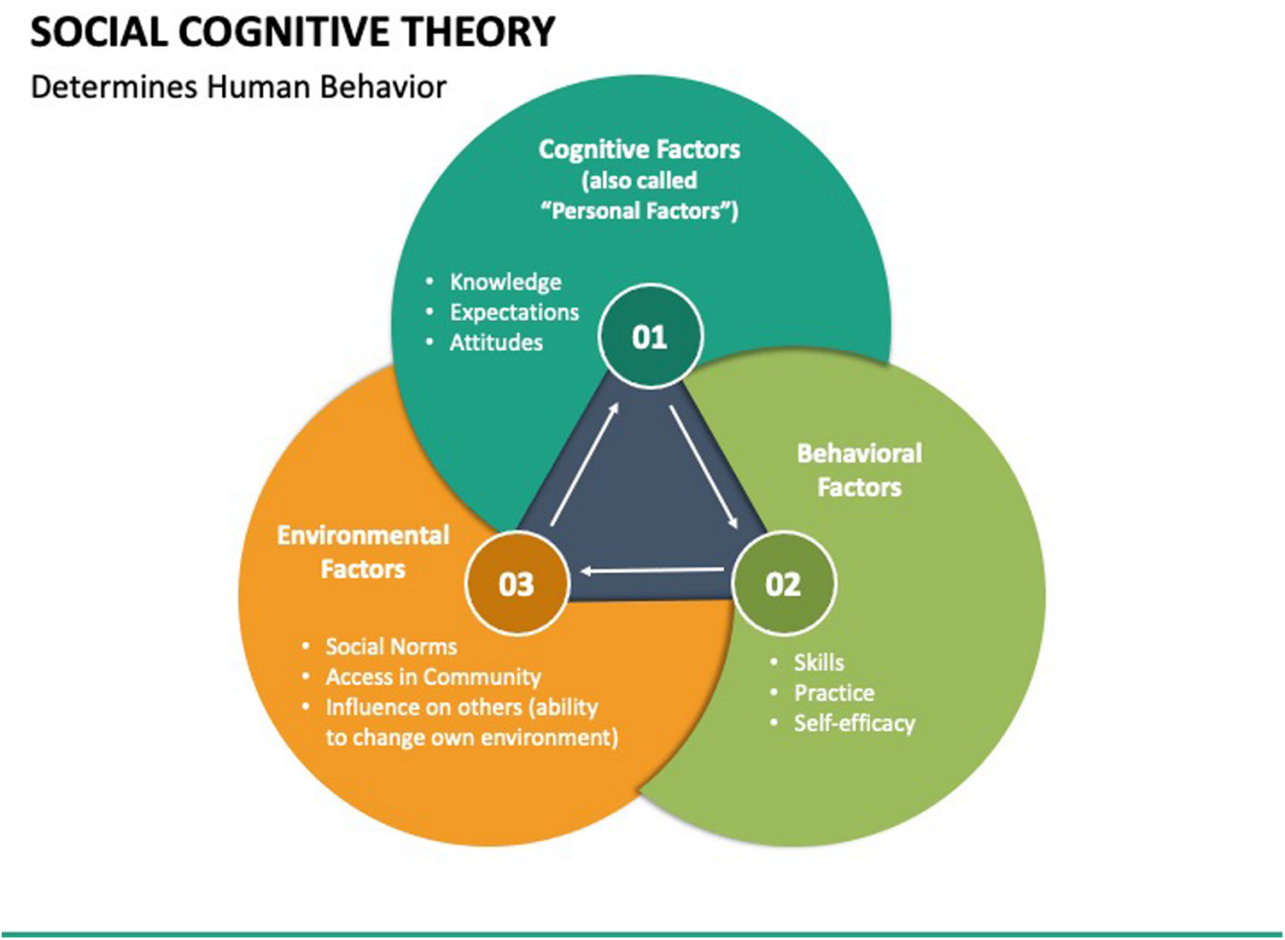
Social cognitive theory framework (33).

**Figure 2:**
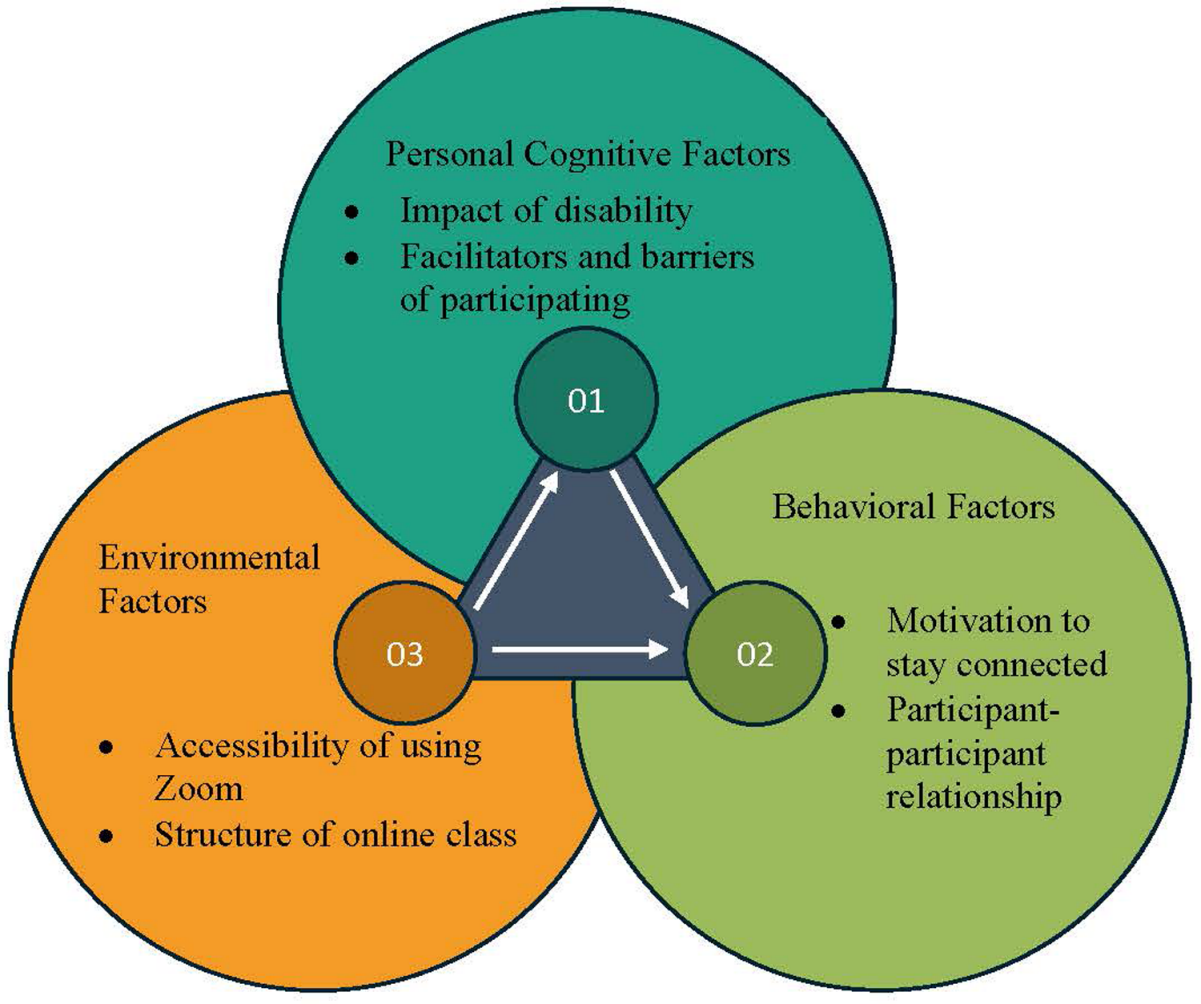
Social cognitive theory as it applies to the current study.

In health promotion programs specifically, SCT analyzes both initiation and maintenance of behavior. It allows participants to regulate their actions by achieving goal-oriented behaviors by learning situational and environmental control, and by reinforcing their learned patterns of control (20–23). Health promotion programs designed using SCT use specific outcome measures to assess readiness for change and implemented change across time. Figure 1 illustrates the determinants of SCT by which participant's behavioral changes are defined and assessed. The MENTOR program uses SCT to encourage positive behavioral change among PWD using a virtual environment by introducing dynamics such as personal attitudes, socioenvironmental influences, and self-efficacy. Figure 2 demonstrates the SCT using constructs specific to the current study.

The interview guide developed for this study employed Dr. Bandura's concept of reciprocal determinism and SCT's constructs (personal cognitive factors, socioenvironmental factors, and behavioral factors) to develop each question. An independent researcher was part of the survey development iteration process to ensure accuracy and content validity. The survey was formatted to assess participant's perceptions of MENTOR during the program as well as their level of implementation following their completion of the program.

### Qualitative approach

A qualitative approach was used in this study to gain a deeper understanding of participants’ retrospective experiences and preferences during and after participating in the MENTOR program. Compared to other methods of data collection, such as surveys, qualitative interviews allow the researcher and participant to have fluid, in-depth conversation to immerse and qualify their perceptions of the exercise portion of the MENTOR program, and how those experiences might have affected their implementation of learned strategies following the program. To best collect this information, our qualitative approach was designed to allow relevant information to be shared between the researcher and participant. Thus, semi-structured, virtual interviews guided by a curated interview guide were employed.

### Data collection

#### Development of interview guide

To increase the rigor and ethics of interviewing individuals with physical disabilities, the primary researcher conducted a focus group panel of expert advisors to review an initial interview guide developed using SCT. Selection criteria for participating in the focus group included: (1) 18 years of age or older; (2) self-identify as having a physical disability/mobility limitation, and/or (3) experience with delivering inclusive/adapted content to people with physical disabilities; (4) able to speak and converse using English language; and (5) willing to provide knowledgeable feedback on readability and inclusive language usage. Five individuals whose expertise included community-based disability programming, disability and physical activity, and disability survey development were identified and invited to attend a focus group session. All five individuals agreed and attended. Following focus group feedback, a final interview guide was developed.

#### Participant interviews

The interview guide was designed as semi-structured to allow participants to speak about their personal experiences. One-on-one virtual, semi-structured interviews were conducted via Zoom between the primary researcher and participant. Participants were informed their participation was voluntary and that they could discontinue their interview at any time. Participants were also informed that audio and visual data were recorded for data analyzation purposes only and were stored using a HIPAA compliant, duo-authenticated database secured by private networks. To make participants feel comfortable, before interviews began, the researcher asked each participant an ice-breaker question of “Tell me a little bit about yourself,”. Once interviews officially began, the researcher used the interview guide as a checklist of questions to ask, while also asking participants to elaborate more on certain aspects of the conversation that were of interest and not listed in the interview guided questions. For example, if a participant shared an interesting detail about the exercise classes not listed in the interview guide, the researcher might ask the participant to continue sharing. The semi-structured design allowed the researcher insight to participant's complete involvements during the MENTOR exercise classes. Per *a priori* protocol agreed upon by the research team, interviews would be conducted until qualitative data saturation occurred and was maintained.

## Data analysis

Qualitative data was recorded using Zoom, transcribed using Microsoft 365 Word transcription service, verified by the primary researcher, and analyzed using NVivo Software (version 14). Data was informed by ontological relativism, which means reality is dependent on the person/people interpreting it. To increase the rigor of data analyzation, two coders (M. M. and C. F.) analyzed data independent of one another, then met to agree and merge data. In the event of coding discrepancy, a third coder (L. B.) was contacted. Interview transcripts were analyzed in sequential order of interviews conducted. Codes were created beginning in the first interview and either added-to or deleted depending on the saturation of relevant data as additional interview transcripts were analyzed. M. M. and C. F. created a qualitative codebook with definitions to remain in tandem when interpreting interview transcripts independently. Where new codes were created, M. M. and C. F. met to agree on the new code, defined it, and inserted it into the codebook. This process was repeated until data saturation occurred. See Appendix 1 for codebook.

Results of qualitative data were emailed to participants for member checking to increase the internal validity and trustworthiness of the study. Based on *a priori* significance agreed upon by the research team, qualitative results were considered significant if 5 ore more participants (63%) reported the same code. Table 1 illustrates participant demographics.

**Table 1:** Participant demographics (*n* = 8)**.**

Participants (*n* = 8)	Mean (SD)
Age (years)	50.4 (13.37)
Disability	
Stroke	62.5% (5/8)
Arthritis	12.5% (1/8)
Multiple sclerosis	12.5% (1/8)
TBI	12.5% (1/8)
Ethnicity	
African American	12.5% (1/8)
Multiracial	12.5% (1/8)
White	75% (6/8)
Geographic location	
California	12.5% (1/8)
New Mexico	12.5% (1/8)
Missouri	12.5% (1/8)
Louisiana	12.5% (1/8)
Alabama	50% (4/8)
Adherence	
Group	83.75% (5.52)
Ppt 1	81%
Ppt 2	75%
Ppt 3	88%
Ppt 4	81%
Ppt 5	94%
Ppt 6	88%
Ppt 7	82%
Ppt 8	81%

## Results

A total of eight high-adherers (*n* = 8) to the MENTOR virtual exercise classes were invited to complete interviews, and 100% of participants consented and completed their interviews. Data saturation occurred at the sixth interview and was maintained through the eighth interview, at which point no more interviews were conducted from the remaining pool of high adherers. Average interview length was 47 min with a range from 34 to 69 min. Thirty-five participant-generated codes were identified. These participant-generated codes were categorized using SCT constructs, personal cognitive factors, socioenvironmental factors, and behavioral factors. These existing constructs were pre-determined as themes to shape the interpretation of participant-generated codes found from interviews. Table 2 reflects interview guide questions and results of coding analysis. Only significant results are presented here, which we defined at an *a priori* value of 63% or more of participant report per code. To increase internal validity and trustworthiness of the results, member checking was employed. All participants were emailed a copy of their transcript as well as comprehensive findings, to which 100% of participants responded in agreement to the data.

**Table 2:** Participant responses (*n* = 8).

Question	SCT themes	Codes	Description
Q1: You finished MENTOR exercise classes with high adherence, which means you attended 12 or more out of 16 exercise classes. What made you decide to attend that many classes?	Personal cognitive Socioenvironmental Behavioral	Reason for high attendance	100% of participants reported subjective reasons for high attendance. • Four participants reported the commitment they made to the program • Two participants reported they liked the disability friendly environment • One participant mentioned they felt better the more they moved • A final participant mentioned they joined to share information they learned from MENTOR with a stroke group
		Reasons for absences	100% of participants noted they had in fact missed one or more classes. • Five participants reported they missed because of a prior commitment they made before MENTOR started • Two participants did not mention a specific reason for missing • One participant reported they were a caretaker for their family member and missed because of this responsibility
		Motivation to stay connected	When probed about their motivation to stay connected to the program after they missed a class(es), • Six participants reported that their motivation to stay connected came from the consistency of classes (33%), other participants (33%), and future program opportunities (33%) • The remaining two participants reported they were motivated by personal medical benefits they perceived they were receiving as a result of participating
		Online environment	When participants were asked about their level of participation within the group and with their online exercise instructor, • Four enjoyed the virtual teaching piece and participant interactions • Three enjoyed the virtual teaching piece but not participant interaction • One did not enjoy the virtual teaching piece or the participant interaction.
		Attitude towards participating	100% of participants mentioned that they were optimistic about their health because of their program participation.
		Subjective level of participation	Five participants also mentioned that while they felt optimistic about their health due to their participation, preferences existed among difficulty of exercise and their vocality about it. • 100% of participants mentioned that they were not vocal during class, either because instruction filled the entire time, or because they felt too shy to ask questions in a group setting • One participant wanted harder exercises • One participant wanted shorter exercise classes and more interaction from instructor • One20% of participant felt the exercises were fairly easy • Two participants felt the exercise difficulty was appropriately presented with options to make them easier or harder depending on what the participant wanted
Q2a: How were your expectations met for disability representation?	Personal cognitive Socioenvironmental	Disability representation	100% of participants felt they were accurately given modifications to match their disability or comfortability for performing exercises within the program. When probed about any preferences participants had that were not addressed or met, all participants stated they did not have any preferences that were left unaddressed or unmet.
Q2b: How were your expectations met for modifying, adapting, or individually tailoring the exercise class material?	Personal cognitive Socioenvironmental	Class material – positive	Five participants talked about liking the class material and how it matched the capabilities and demands of their bodies while being physically active. Specific examples include the accessibility of the physical movements, adaptations offered, and the appropriate length of class time.
		Exercise instructor feedback – positive	Additionally, five participants mentioned their preferences for the exercise instructor and the structure of the online exercise group. Specifically noted was that they felt the instructor was knowledgeable and always offered fair and timely suggestions to modify exercises being done during class.
		One-on-one session	When probed about scheduling a one-on-one session between the participant and the instructor, five participants responded • Four participants reported they did not feel like they needed an individual appointment • One mentioned speaking with the exercise instructor about a disability-specific exercise question
Q2c: How were your expectations met for the online interaction component?	Personal cognitive Socioenvironmental	Class structure	100% of participants mentioned they had no expectations entering the program. Opinions differed slightly, however, among participants regarding the level of interaction between their class and the instructor. • One participant mentioned not much group interaction other than receiving exercise-specific instruction • Another participant mentioned not liking the way the instructor gave critiques to the group, which lessened her perspective of high expectations for the rest of her time within the exercise classes • A different participant noted specifically that their other classmates served as motivation for him to return to classes • Two participants mentioned they thought their group interacted well amongst each other and with the exercise instructor
Q3: Do you feel the virtual exercise class benefitted you?	Personal cognitive	Benefits of participating	All participants mentioned benefits of participating: • 63% mentioning facilitators of participating • Examples of facilitators include receiving exercise equipment that they kept even after the program is over, class schedule consistency, and a passionate staff who enjoyed helping • 86% mentioning the impact that exercising had on their physical and mental well-being • Examples of exercise impact include participants being made aware that despite their physical disability, they can still participate in physical fitness; learning new exercises to replace sedentary time; and learning how to listen to your body, according to participants
Q4: What perceived facilitators existed for you during your time within the MENTOR exercise classes?	Personal cognitive Socioenvironmental Behavioral	Facilitators to participating	All participants reported facilitators to participating. • Specific examples include the tailored exercise class curriculum, the exercise instructor, the exercise equipment, socialization, gaining confidence, the use of Zoom to deliver the program, and having camaraderie among participants.
Q5: What perceived barriers or difficulties existed for you during your time within the MENTOR exercise classes?	Personal cognitive Socioenvironmental Behavioral	Barriers to participating	75% of participants mentioned experiencing barriers for their time within the program. • Specific barriers included their own mindset being a barrier to overcome to fully participate, the impact of participant's disabilities like mental processing, doctor's appointments being scheduled at the same time as class, limited physical space at home to fully exercise, fatigue after exercise class, and the attitudes of other participants. • Two participants mentioned experiencing no barriers.
Q6: What would you change about the program if anything?	Personal cognitive Socioenvironmental Behavioral	Program changes	When participants were asked what they would like to change about the program, • Three participants wanted an accountability club for after the exercise classes ended to stay connected to their group and what they learned during the eight weeks • One participant mentioned wanting more interaction from the instructor during the classes • Another participant wanted scheduled makeup classes during the week • Another three participants noted they would not change anything about the program Participants were probed about their performance or attendance within the program if their support network were to join them in classes. • All participants said their support network being present would not influence their performance or attendance; however, • Four reported they would not prefer their support network to be involved mentioning specifically they liked the allocated time alone to be social with new acquaintances and enjoyed the independency • Three reported they would like their support network to be involved • One reported they are indifferent to the idea
Q7: Would you recommend this program to others?	Socioenvironmental	Recommending MENTOR to others	When participants were asked if they would recommend the MENTOR program to others, it was a unanimous report that all participants would recommend this program to others. In fact, some participants mentioned that they had already recommended it to friends, family, or colleagues or helped a friend, family member, or colleague get signed up for the program.
Q8: Relationships with online instructors are described as your comfort level in speaking and interacting with them during the program. Considering this, what was your relationship like with your online exercise instructor?	Personal cognitive Socioenvironmental Behavioral	Exercise instructor	Six participants reported having a great relationship with their online exercise instructor. • Specific examples include receiving modifications for their disability during class, benefitting from the instructor's knowledge, being motivated to work out with a coach who enjoyed working out themself, and having fun in the exercise classes. • Critiques about the exercise classes include the instructor lingering in lecture for too long (25%), using advanced and technical vocabulary that was difficult to understand (38%), and using videos too much during class instead of performing the exercises himself (12%).
Q9: What was your relationship like with the online exercise group, the other participants?	Personal cognitive Socioenvironmental Behavioral	Participant-participant relationship	• One participant expressed his commitment to his fellow participants and was motivated by them throughout the classes • Two participants specifically mentioned a sense of camaraderie between their groups and expressed enjoyment • five participants stated they had little to no interaction between the group and wished there were more opportunities to speak and be social
Q10: What motivated you to stay involved in the online exercise classes?	Personal cognitive Socioenvironmental Behavioral	Motivation to stay connected	All participants mentioned personal and specific reasons that they were motivated to stay connected to the program. • Three participants mentioned their internal drive to get better and continue their recovery. • Other participants mentioned similar reasons such as they found motivation from their instructor and fellow participants (25%), and the commitment they made to become more physically active (12%). • Two participants specifically mentioned that the online exercise classes alleviated the barrier of driving and going into a gym.
Q11: Zoom fatigue or zoom exhaustion is described as feeling tired by the effort of being on Zoom to participate. Knowing this, how would you describe your relationship with Zoom fatigue during your time in the MENTOR program?	Socioenvironmental	Disagrees – likes Zoom	100% of participants reported that they never experienced feeling exhausted by using Zoom to participate virtually.
Q12: What else would you like to say about the online exercise classes?		Benefits of participating, class material – positive	When participants were asked if there was anything else they’d like to say about the program, each participant reiterated that they benefitted from the exercise classes and were fond of the class material. • Specific items that were mentioned include learning valuable information pertaining to disability and exercising, having recorded exercise videos as resources to use even after the program completed, regaining confidence, accessible, and being able to keep their exercise equipment to use after the program completed.

Table 2 provides participant responses to each of 12 interview questions. Overall, participants described the exercise classes as motivating, disability friendly, easily adaptable when questioned about their involvement during classes. Outside of the confines of the interview questions, 100% of participants mentioned still being physically-active today using MENTOR exercise-specific content including recorded videos, exercise equipment, and communication with fellow participants they keep in contact with since their time in the program ended. When asked about their perception of the virtual exercise program structure, participants reported benefits of participating, more facilitators than barriers, a positive relationship with their virtual exercise instructor, and willingness to recommend the program with others. Additionally, 100% of participants informed us they never experienced Zoom fatigue or exhaustion of participating in weekly classes online.

Table 3 highlights the overarching quotes that participants made about the exercise classes. Overall, participants expressed great satisfaction and gratitude for this type of program existing for PWD, reasons they found motivation to continue with high attendance, and the impact MENTOR had on them personally.

**Table 3:** Selected participant quotes.

Codes	Exemplary quote
Motivation to stay connected	“Well, number one was a sense of obligation. Like, I mean, I felt like I committed to this thing and I was obligated to finish it. I would also say because I liked the instructor and I liked the folks that were in the program with me and so I wanted to join them and be a part of it.” – Ppt 1
Barriers to participating	“I mean during the sessions, of course, you know it's kind of awkward in some places because it's Zoom and you can’t really do the movements or you’ve got to get a piece of equipment, so you’ve gotta get that piece of equipment, so that was a little strange.” – Ppt 1
Reasons for high attendance, attitudes toward participating	“The demonstrated efficacy of the programs I’ve done through NCHPAD so far, I mean, you know, if I find it works, I’m inclined to stick with going with what works.” – Ppt 2
Disability representation, class material – positive	“…I felt that I could most of the time do it either way, whether it was totally modified, you know or standing, sitting, however they wanted to do it I was fine with that. But I was glad there was an option to contact the instructor and get individual help, so yeah, I would say that was a big plus there.” – Ppt 3
Program changes	“The exercise, that's tough because I felt like two days a week was good, but sometimes it’s difficult whether from being tired, or maybe that was a high pain day or you know whatever. And I know those are barriers in a way, but that would be the only thing I wish there was like a makeup class, you know that you could join.” – Ppt 3
Online environment	“I’m not ready to just be sitting around, so that's why I attended so many classes. It was fun. I got to talk with people, like, you know, we got to listen to each other's experiences.” – Ppt 4
Exercise instructor – positive feedback, motivation to stay connected	“It was the staff, the people that led the classes, they were compassionate, they were into what they were doing. They seem to really enjoy helping us, the really seem to enjoy what they did. They seemed like they had a sense of purpose about what they were doing and that made me want to come back because the instructors were nice…they didn’t make you feel like you gotta do this and you’re not gonna get anywhere if you don’t do it.” – Ppt 4
Benefits of participating, disability representation	“…I thought it was a really good program and I would recommend it to anybody, especially with a disability, you because you don’t know, you know, or you think you can’t do something and then it teaches you that you can do so much more than you thought.” – Ppt 5
Class material – negative	“He spent most of the class just talking and demonstrating, so maybe taking some time out and saying, you know, does anybody have any questions or you know, make it more talkative in the group so that people in the group can interact more…” – Ppt 5
Subjective level of participation	“Exercise I felt the most fatigue after, but that was just because I was, like moving my body and afterwards I just needed to sit and breathe and let myself, I was so exhausted after it.” – Ppt 6
Disagrees, likes Zoom	“I did not have any problems with it. Like I said before, I’m very visual, so I think it helped having the, you know, the live action, having someone there, doing the exercises with you, not just speaking to you and then starting a video.” – Ppt 7
Class structure	“But the way it came across, all the classes adapted things well. So he found he could take away something from all the classes, I felt like he… and the way they were broken up throughout the week was nice. You know that gave him something to look forward to.” – Ppt 8a

NCHPAD, National Center for Health, Physical Activity and Disability. Ppt 8a represents caretaker for Ppt 8 whose speech was affected by stroke.

## Discussion

This study was a qualitative evaluation of highly adherent participants who participated in an eight-week virtual wellness program that included two days a week of exercise classes. The purpose of evaluating this sample of PWD was to analyze how their adherence affected their participation in and perception of the program. Results suggest that the eight-week online exercise classes were effectively received for those participants who maintained high adherence. The thematic coding of the participant interviews produced positive findings about the virtual exercise classes, signifying that participants had an overall positive experience with minor critiques regarding their perception of program delivery. Moreover, participants expressed comfort with the online atmosphere and interactions among their instructor and co-participants. These follow-up interviews ranging from seven months to twenty-two months post-program completion found that 100% of participants were still maintaining their exercise regimens, using the program-provided exercise equipment, and utilizing the recorded exercise videos as a resource to continue physical activity.

The use of SCT deepened our understanding of how participants perceived integrating a virtual exercise program into their life. Personal cognitive factors that applied to our sample included outcome expectations, knowledge, and behavioral capability. Participants stated in several instances they used MENTOR for their own medical benefit. This signified their readiness for engaging in self-managed personal health. Socioenvironmental factors that affected participant's perceptions and behaviors during and after MENTOR exercise included the mandatory use of Zoom to interact and participate. While participants were unbothered by the time they spent on Zoom, regarding class structure, participants expressed preferences such as more opportunities to socialize outside of instruction time (*n* = 7) and more group-facilitated interactions compared to instructor-led lecture (*n* = 6). Additionally, they noted certain facilitators and barriers of virtual participation. Facilitators included the tailored exercise class curriculum, the exercise instructor, the exercise equipment, gaining confidence, the use of Zoom to deliver the program, and having camaraderie among participants. Barriers included mindsets to overcome to fully participate, the impact of participant's disabilities (i.e., mental processing), doctor's appointments being scheduled at the same time as class, limited physical space at home to fully exercise, fatigue after exercising, and the attitudes of other participants.

Finally, behavioral factors that were noted by the participants were self-regulatory practices such as skill, intensity, and self-monitoring which includes goal setting, planning, and awareness of one's health. All participants consistently mentioned the effect exercise had on their health while they were in classes. They also learned how to adapt and modify exercises to fit their personal needs related to their function and disability. All participants noted the impact that learning through a disability-friendly environment had on motivating them to maintain and implement what they learned from the MENTOR exercise classes into their daily lives.

To our knowledge, there are currently no other accessible, virtual holistic-based health and wellness programs offered specifically for PWD that target comprehensive areas of health such as mindfulness, exercise, and nutrition (3, 25, 34). As mentioned by participants, this program alleviated barriers to participation and included facilitators that allowed them to successfully participate and implement what they learned into their lifestyles after they completed the program.

A few post-evaluation recommendations were noted by participants. First, future iterations of MENTOR should incorporate participant feedback to the exercise instructor while the program is ongoing so that barriers can be removed as soon as they are identified. Second, seven out of eight participants (88%) desired more interaction between their group and their instructor, less intimidating vocabulary, and less lecture format with a limited number of pre-recorded videos played during class time. Third, participants noted that a specific barrier was not having their room and equipment ready before the start of class. They recommended that the instructor should let them know in advance which exercises were going to be performed before class so that the participant could have an appropriate amount of space and the correct equipment prior to the start of class.

A 2011 study that surveyed PWD identified important factors to make them want to participate in physical activity, including accessible exercise facilities, instructors knowledgeable about adapted exercise, a sense of belonging, support from friends and family, and disability awareness (35). MENTOR circumvents and accommodates these variables by offering a free, completely tailored online program for different types of disabilities, age groups, races, and geographic locations. The most reported reasons PWD have given when asked why they discontinue exercise are inaccessibility, fear, and the fatigue that follows physical exertion (36–39). These barriers can be overcome with personnel trained in disability fitness, inclusivity, and accessibility (38). Currently, blueprints for improving exercise adherence now exist so that future studies can shift from reporting facilitators and barriers to developing strategies and interventions that address reported barriers and use key facilitators to promote adherence (40–42). Furthermore, more interventions specific to PWD are incorporating theories and frameworks, which allows data to be empirical and clinically translatable (35, 36, 43). The aforementioned strategies allude to the continued need of health promotion to, for, and among PWD, especially targeting holistic wellness.

This study had limitations. First, we intentionally developed an interview guide specifically for the high adherent completers of MENTOR exercise classes. A more complete profile of adherence may have been obtained if interviews were conducted with non-, low-, and moderate-adherers. Second, the majority of participants were stroke survivors over the age of fifty and could have perceived exercise differently than other participants. Third, data analyzation included the primary researcher and a secondary independent researcher, which may have introduced bias, whereas a coder not affiliated with the interviews might have different impressions of the data. Finally, only exercise classes were used for interview purposes considering the scope of our aim. To fully understand the comprehensibility of the full program, interviews should be conducted among all core program classes to incorporate mindfulness and nutrition. Future delivery of MENTOR should incorporate feedback from participants who completed the program with high adherence, and more independent interviews should be completed within all areas including mindfulness and nutrition. Nonetheless, the data illustrate the theoretical factors associated with a successful online exercise program in a small group PWD with a variety of races, ages, and geographic locations.

## Conclusions

This qualitative study guided by the SCT framework provided an insightful examination regarding high adherent participants within the MENTOR exercise classes and their perception of the program delivery. MENTOR exercise classes produced efficient and effective results for participants who were high adherers to the program. We found participants (1) were motivated to stay connected, (2) self-reported benefits of participating in the program, (3) felt represented regarding their disabilities, (4) would recommend the program to others, and (5) enjoyed their instructor, the class material, their fellow participants, and the virtual delivery. These results also included suggestions, feedback, and recommendations including (1) more instructor interaction during class, (2) less lecture time and format from instructor, 3) prior knowledge about what exercises participants would be performing and with what equipment, and 4) scheduled make-up classes for missed classes due to prior commitments. These results provide MENTOR personnel with guidelines to support future iterations of the program from a successful participant's perspective, as well as a beneficial framework for other exercise-promoting programs.

## Data Availability

The original contributions presented in the study are included in the article/Supplementary Material, further inquiries can be directed to the corresponding author.

## Appendix 1

**Table:** Qualitative codebook used for data analysis between coders.

CODES	MENTORExQual
	Description
Benefits of participating	Perceived benefits from participating in MENTOR program and/or exercise classes
Class material	Any comments related to exercise prescription
Knowledge gained	Key takeaways regarding exercise and/or disability
Negative - class material	
Positive - class material	
Comparisons to co-participants	Degree of assessing other participants within exercises classes
Disability representation	Inclusivity of varying disabilities and providing accurate adaptations within exercise classes
Exercise equipment - NEW CODE	Any comments (positive or negative) related to exercise equipment provided by MENTOR Program
Exercise impact	Physical and mental results from participating in exercise class
Exercise instructor feedback	Any comments related to exercise instructor - anything that John says or provides during class (ex. adaptations)
Negative feedback	Feedback to improve instruction
Positive feedback	Any positive comments on exercise instructor
Impact of disability	Disability Contribution to Being Physically Active
Level of participation	
Attitudes towards Participation	Mindset entering, during, and after program - including anticipated ability to perform exercises
Barriers to participation	
Facilitators to participation	
Subjective level of participation	Participants’ opinions on their level of participation within exercise classes
Motivation to stay connected	Positive influences related to exercise class attendance
Program produced motivators	External motivators (Facilitated within MENTOR) to participate in classes. Examples - instructor, co-participants, equipment box
One on one session	
Online environment	Any comments related to nature of virtual classes - Ex. Set up, Zoom connection, communication with instructor/co-participants during classes
Online interaction	Subjective peception of interactions with class - Ex. Class requirements, Preparedness, Expectations
Participant desires	Any comments of wants or needs outside of direct questioning of program changes
Participant-participant relationship	
Perception of disability	Individualized/inter-personal perspective regarding one's own abilities/disabilities
Physical activity enjoyment	
Identity - physically active	
Program changes	Related to specific changes of what they would change in MENTOR program
Reason for high attendance	
Reasons for absences	
Recommending MENTOR to others	Whether or not they would recommend program externally
Social support opinion	Perception of including members of external support network within MENTOR program
Zoom fatigue	Opinions of time requirement spent on Zoom for program
Disagrees - likes Zoom	
